# Factors influencing the nature of client complaint behaviour in the aftermath of adverse events

**DOI:** 10.1002/vetr.4966

**Published:** 2024-12-29

**Authors:** Julie Gibson, Kate White, Liz Mossop, Marnie L. Brennan

**Affiliations:** ^1^ School of Veterinary Medicine and Science University of Nottingham, Sutton Bonington Campus Loughborough UK; ^2^ Centre for Evidence‐Based Veterinary Medicine University of Nottingham, Sutton Bonington Campus Loughborough UK; ^3^ Sheffield Hallam University, City Campus Sheffield UK

## Abstract

**Background:**

Negative veterinary client complaint behaviour poses wellbeing and reputational risks. Adverse events are one source of complaint. Identifying factors that influence adverse event‐related complaint behaviour is key to mitigating detrimental consequences and harnessing information that can be used to improve service quality, patient safety and business sustainability.

**Methods:**

Interviews were conducted with five veterinary client complainants and five veterinary client mediators. Qualitative content analysis of the transcripts was used to identify categories of capability, opportunity and motivation influencing client behaviour. One category of motivation identified focused on the desired outcomes of complainants. Two hundred and eighty resolved veterinary‒client mediation cases related to adverse events subsequently underwent content analysis to quantify these desired outcomes.

**Results:**

Client complaint behaviour was motivated by clients’ emotional reactions, perceptions and beliefs and desire to achieve an outcome as a result, and was influenced by previous complaint experience, technological ability, self‐confidence and broader organisational and societal factors. Although financial redress was the most commonly identified desired outcome, apology, honesty, accountability and prevention of future events were valued.

**Limitations:**

Small data sets and interpretative analyses limit the generalisability of the findings.

**Conclusions:**

Proactively engaging clients in relation to adverse events is likely to reduce negative complaint behaviour and facilitate veterinary quality improvement.

## INTRODUCTION

Harms caused by healthcare provision, rather than being suffered as a consequence of underlying disease, are defined as adverse events.^1^ Adverse events represent a source of real or perceived ‘service failure’,^2^, ^3^ regardless of the degree of detrimental impact incurred as a result. Complaint behaviours associated with such events are defined by individuals’ communication of dissatisfaction to perceived influential parties.^4^ These parties include existing or potential clientele but may also encompass other stakeholders. For example, individuals and organisations with a financial interest in the service may be targeted, such as employees, business owners, investors, shareholders and insurers. Complaint behaviour may also be focused directly towards regulators, who may be perceived to be most influential in operationalising action in favour of the complainant.

Complaint behaviours present varying threats to individuals and organisations depending on their type.^5^ Voicer, irate and activist behaviours are associated with sequentially heightened engagement in activities that relay expressions of disgruntlement. They can include written and verbal communications as well as actions. Examples include negative word‐of‐mouth,^6^ negative posting on social media sites,^7^ campaigning in favour of alternative providers and involving external adversarial parties. Such behaviours can be reputationally damaging, with direct consequences for business performance,^8^ and personally damaging for the individuals involved. With passive behaviours, complainants do not openly display concerns but may instead experience an emotionally detrimental and cumulative sense of internal dissatisfaction. This manifests as a gradual disengagement from the current provider and an eventual switch to an alternative without explanation. Passive complaint behaviour is as much of a threat as expressive types of complaint behaviour because it denies organisations the opportunity to learn from events and to reassure and retain consumers in the face of concerns.

Devastating personal, professional and organisational consequences are recognised in relation to human healthcare complaints.^9^, ^10^, ^11^ Healthcare organisations recognise the need to safeguard practitioner wellbeing, patient safety and the sustainability of care. In pursuit of this, they focus on preventing the pursuance of negligence claims and maintaining and restoring relationships between care receivers and providers. Complaint management is embraced as a key aspect of both healthcare quality improvement, which is focused on optimising processes to offer better patient outcomes,^12^ and successful service recovery, which is defined by re‐establishment of complainants’ trust.^13^, ^14^ Cataloguing complaints to detect early patient safety risks^15^, ^16^, ^17^ and tracking complaint trends that can be used both to lever positive systems change and for benchmarking purposes^18^ are established components of complaint management processes. However, it is widely accepted that engaging patients and family in relation to their concerns fundamentally influences the success of complaint management strategies that improve patient safety.^19^ To constructively engage those who are dissatisfied with care, an understanding of factors that influence their complaint behaviour is needed.

NHS Resolution is a body of the Department of Health and Social Care that provides expertise to the United Kingdom's National Health Service on resolving medical disputes and using learnings for enhanced patient safety. In a recent survey conducted by the department, 79% of claimant respondents indicated that one of their reasons for pursuing a claim of negligence was to receive a detailed explanation of the incident.^20^ Financial compensation was stated as the primary reason for making a claim in only 6% of cases. This suggests that claims in human healthcare are predominantly underpinned by perceptions of a lack of transparency surrounding events rather than a desire for monetary redress. Ironically, fear of legal action and associated emotional and reputational repercussions may prevent practitioners from openly disclosing information to patients and families in the aftermath of an adverse event.^21^ Such defensiveness not only compounds litigious drive but impedes efforts to improve the safety and quality of future care.

Alternative dispute resolution (ADR) refers to an array of different methods. All aim to reduce litigious action from complainants and therefore mitigate any associated emotional, reputational and financial consequences.^22^, ^23^ Early disclosure and apology programmes, mediation, arbitration and pretrial screenings are forms of ADR. ADR is institutionalised within human healthcare^24^ and is a recognised component of patient safety agendas.^25^, ^26^ Mediation is a type of ADR where opposed parties attempt to come to their own shared agreed solution regarding grievances. Unlike civil litigation, solutions can extend beyond economic settlements to include apologies, explanations and reassurances that the same will not happen in the future. The mediation process is usually relatively informal, inexpensive, conducted outside of the courtroom and facilitated by an impartial third party who is specifically trained in mediation. Resolution by mediation is associated with high levels of satisfaction, particularly when entered into voluntarily, as any outcomes are agreed by both parties.^27^ The process is non‐binding, meaning that both parties reserve the right to withdraw from negotiations prior to an agreed resolution being reached. Despite the benefits outlined above, mediation may not be favoured by either party as it may not provide a sense of retribution for those aggrieved or a chance for the accused to ‘clear their name’.

As consumers, veterinary clients have a right to a fair resolution of legitimate grievances when adverse events occur.^28^ However, the sector is thought to be increasingly litigious,^29^ and some respondents in the 2019 Royal College of Veterinary Surgeons (RCVS) survey of the professions clearly reported suffering from the ill effects of associated malevolent client behaviour.^30^ As in human healthcare, negative complaint behaviour associated with adverse events jeopardises the wellbeing and professional functionality of veterinary practitioners at whom such behaviour is directed.^31^, ^32^, ^33^ It likewise poses reputational risks and ultimately impacts veterinary care safety and quality. Risk factors for complaint^34^, ^35^ and practitioners’ perceptions and experiences of client complaint behaviour are underscored within existing literature.^32^, ^33^, ^36^ Despite this, an understanding of factors influencing the nature of clients’ complaint behaviour in the aftermath of veterinary adverse events is lacking. Such understanding is necessary to inform client complaint behaviour change strategies. These should aim to reduce emotionally detrimental complaint behaviours, including those associated with costly financial and reputational adversarial actions. Equally, they should direct behaviours that are conducive to quality improvement and service recovery. This study aims to explore factors that influence veterinary client complaint behaviour in the aftermath of adverse events and ultimately address the following questions: (1) What capabilities, opportunities and motivations influence client complaint behaviour in the aftermath of adverse events? (2) What do veterinary clients most commonly desire as an outcome of complaint behaviour in the aftermath of adverse events?

## METHODOLOGY

**FIGURE 1:**
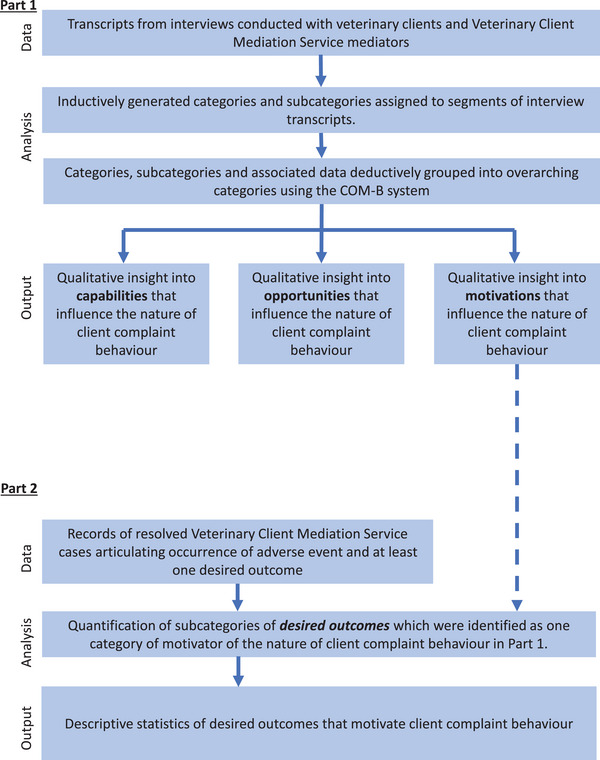
Diagrammatic overview of the research process employed to explore factors that influence the nature of client complaint behaviour in the aftermath of adverse events

Two sequential parts of the study were undertaken and drew from two separate data sources (Figure 1). A content analysis approach was taken, with findings from the first part iteratively informing analysis in the second part. In part 1, interviews with veterinary clients and professional veterinary client mediators were conducted and qualitatively analysed to create categories and subcategories of factors that influence the nature of client complaint behaviour in the aftermath of adverse events. In part 2, data from veterinary client mediation case records were analysed to quantify subcategories of one specific category assessed in part 1. This was done to make inferences about the desired outcomes that most commonly motivate clients who complain in the aftermath of adverse events.

### Part 1: Interviews with veterinary clients and veterinary client mediators

#### Sampling and recruitment

Recruitment of interviewees took place between 1 March and 9 May 2023. A non‐probability convenience and snowballing technique^37^ was used to recruit a sample of five veterinary clients. Veterinary clients with experience of owning an animal (or animals) that they perceived had incurred an adverse event as a result of veterinary care were eligible for inclusion. An adverse event was defined as any unintended physical consequence of veterinary care, including events believed by the client to be the result of a complication, error, mistake or negligence during diagnosis or treatment. As an experienced veterinary surgeon and PhD student, the primary author (J.G.) first invited an eligible client known to her personally to interview. The client was asked to invite acquaintances eligible for inclusion to contact the primary author if they were willing to participate, and J.G. did not have a prior existing relationship with any further participants.

Subsequently, purposeful sampling^37^ was used to recruit five veterinary client mediators. The aim was to gain insight from an impartial party with a broad understanding of the nature of veterinary clients’ complaint behaviour. Veterinary client mediators employed at the Veterinary Client Mediation Service (VCMS) were eligible for inclusion. VCMS mediators facilitate ADR between veterinary practitioners and their clients. VCMS is funded by the veterinary professions’ regulatory body, the RCVS, but is independently provided by a law firm with specialisation in mediation of regulated professions. The RCVS directly refers clients who raise concerns that fall outside their remit, and they also advertise the service through their website.^38^ Veterinary practitioners or their clients can make first contact with VCMS. Participation is voluntary and both parties must mutually agree to enter the mediation process. The trained mediators impartially facilitate veterinary clients and practices to come to an agreement on any issues where opinions differ. Mediators who had not had correspondence with the clients interviewed in the study but who had extensive experience of liaising with clients who had complained in the aftermath of adverse events were included. Recruitment of the mediators involved J.G. initiating contact with the head of VCMS via email correspondence, who then facilitated contact with further eligible VCMS mediators.

#### Data collection

One interview per client was conducted at a public location of their choice and started with J.G. encouraging participants to recall personal experiences of a veterinary adverse event. The purpose of this ‘pre‐questioning phase’ was to establish trust and engender rapport with the participants. A semi‐structured interview approach was then used to focus discussion o complaint behaviour in the aftermath of adverse events.

One interview per veterinary client mediator was conducted at the VCMS head office. A modified version of the client interview guide was used. Modifications were limited to word changes that made questions relevant to mediators rather than clients and sought to expand and clarify any uncertainties in interpretation following the client interviews.

An active listening^39^ approach was used throughout all interviews with the view to gathering rich insights.

#### Ethical considerations

Ethical approval was granted for this interview study by the Committee on Animal and Research Ethics (CARE), School of Veterinary Medicine and Science (SVMS), University of Nottingham (UoN) (ethical approval numbers 2444 180724 [mediators] and 3790 230215 [clients]). All interviews were conducted, recorded and transcribed using the Microsoft Teams video conferencing platform by the primary researcher (J.G.). Positionality and ‘insiderness’^40^ had ethical implications during the interviews. J.G.’s status as an experienced veterinary practitioner (with prior acquaintance with one participant) may have introduced asymmetric power relations and influenced participants’ disclosures surrounding certain events and perspectives. In addition, the sensitive nature of the research had the potential to impact the researcher, the participants and the relationship between the two. To help navigate these ethical complexities, J.G. employed a reflexive approach, particularly to self‐disclosure. The participants were given written information about the study, provided explicit signed consent prior to the interview and were made aware that signposting to support could be provided if necessary. Only J.G. had access to participants’ personal data, which were deleted immediately after completion of the interview. Following the download of the transcribed files, videos and transcriptions were deleted from the Teams site. J.G. removed identifying information from the transcribed files and stored them in a redacted format in line with the UoN's general data protection, research data management (RDM) and data secure data handling policies.

#### Data analysis

Qualitative content analysis^41^, ^42^ was conducted to identify factors that influence clients’ complaint behaviours. The interview transcripts were read twice by J.G. in a process of data familiarisation prior to analysis, which utilised Microsoft Excel^43^ to facilitate category development. Segments of transcript were inductively grouped into categories of similar meaning. A manifest approach was taken. Categories assigned aligned with the ‘obvious’ meaning of the data, in contrast to latent content analysis^44^ or reflexive thematic analysis^45^ where researchers look for hidden depth. When these categories had a shared meaning, they became subcategories under a newly named category.

A deductive approach, which harnesses existing theory to focus research findings and facilitates the determination of relationships between categories,^46^ was then employed to group the categories and associated data. The capability, opportunity and motivation of behaviours (COM‐B) system lies at the centre of the behaviour change wheel^47^ and is a theoretical framework for designing behaviour change interventions. The system reflects that these three conditions interact to generate particular behaviours, which then feedback to influence the continued generation of the behaviour. Capability is inherent to individuals and is defined by their capacity to cognitively and physically engage in a behaviour. Opportunity comprises factors external to the individual that impede, prompt or facilitate the behaviour and includes physical, situational and social means. Motivation refers to cognitive, habitual and emotional processes that catalyse and direct behaviour and is itself influenced by capability and opportunity. Categories, subcategories from which they were derived and associated data were assigned into capabilities, opportunities and motivations that influence the nature of client complaint behaviours.

### Part 2: Retrospective review of VCMS case records

#### Sampling

The basic details of every case dealt with by VCMS mediators are recorded in a standardised format for reporting and analysis purposes. Stored records contain information about the species of animal to which the case relates and free text summaries written by VCMS mediators regarding the nature of the dispute, the desired outcome of the client and if and what resolution was finally reached through mediation. Cases are also categorised according to the type of issue to which they relate. Cases relating to real or perceived unexpected or unintended patient outcomes or ‘adverse events’ are assigned to a category named ‘standard of care’.

#### Data collection

VCMS granted the primary researcher (J.G.) full access to all case records dating from 2 February 2022 to 9 May 2023 that (1) were categorised by VCMS mediators as ‘standard of care’ issues and (2) reached eventual resolution through mediation. J.G. reviewed the records, and those interpreted as (1) failing to articulate the occurrence of adverse event(s) or (2) lacking information regarding clients’ desired outcomes were excluded.

#### Data analysis

One category identified in part 1 as a motivator of veterinary clients’ complaint behaviour related to the outcome(s) they desired in the aftermath of adverse events. To make inferences about the frequency of desired outcomes of veterinary client complainants in the aftermath of adverse events, a quantitative approach was taken.^48^ J.G. read the VCMS mediator's free text summary describing the ‘desired outcome’ of each VCMS case collected and assigned it to the relevant subcategory of desired outcome created from analysis of interview data from part 1. Descriptive statistics using proportions and percentages for each subcategory were then calculated.

#### Ethical considerations of data collection and analysis

Ethical approval was granted for the data analysis phase of the work by CARE, SVMS UoN (ethical approval number 3892 230725). Stored VCMS records do not purposefully contain information regarding those participating and the negotiation process. Although such details are needed during the process, they are destroyed once completed. In addition, VCMS records were screened and any identifying information was removed by a VCMS mediator prior to allowing J.G. access. Records were transferred from VCMS to J.G. electronically using a password‐protected file link, and J.G. downloaded and stored the file in line with UoN's RDM policies.

## RESULTS

### Part 1: Qualitatively derived factors that influence the nature of veterinary client complaint behaviours in the aftermath of adverse events

A total of 10 interviews were conducted: five with veterinary clients and five with mediators employed at VCMS. All veterinary clients and veterinary client mediators having initial contact with the researcher participated in an interview and none withdrew consent following participation. Interviews with veterinary clients ranged from 27 to 59 minutes in duration. Interviews with veterinary client mediators ranged from 24 to 38 minutes in duration. The categories, subcategories and example quotes derived during content analysis using the COM‐B framework are summarised in Table 1. All of the participants discussed at least one capability, opportunity and motivation.

**TABLE 1:** Categories, subcategories and exemplar quotes derived from the interview transcripts using the capability, opportunity and motivation of behaviours (COM‐B) system to guide content analysis

Overarching COM‐B category	Category	Subcategory	Exemplar quotes
Capability: cognitive and physical capacity to engage in the behaviour	Experience of complaints	**‒**	‘Sometimes people know what to do because they have done it before!’ (Mediator 5) ‘I work for the NHS so I am used to complaint procedures. I know my way around a complaint so that helped to … to you know well know how to go about it all’ (Client 1)
	Technological factors	**‒**	‘I do think it makes it easier nowadays cos you can be in touch about it with all the connections there are … it's a click of a button and I don't even need to speak to anyone. Quick and easy’ (Client 1) ‘… everything is online, doing this or that by email … it [making the complaint] was difficult, confusing’ (Client 3)
	Self‐confidence	**‒**	‘some are actually very nervous and need a helping hand as they feel like they don't, well, … they don't feel they are able to properly say what they want to say’ (Mediator 4)
Opportunity: prompts and facilitators of the behaviour	Social and societal factors	Availability of information	‘… their issues have often just happened and sometimes we get people phone us from the car park because they're just at a loss, they don't know where to turn to … um … and they may sort of jump onto Google and type in “How do I complain about my vet?” And you know that that's when we [VCMS] come up. I've noticed that sort of fairly recently that they just seem to do it as a first line’. (Mediator 1) ‘We're bombarded with stuff now, no win no fee, then you have people seeing what happened and saying you know you should be compensated for that’ (Client 4)
		Social networks	‘I knew someone through [online discussion forum] who'd been through similar, they reached out at first helped me with how to go about it [the complaint]’ (Client 5)
	Organisational factors	Processes within practices	‘It said on the [practice] website that I could complain but that felt too much, too formal, like … like … I didn't have time …’ (Client 3) ‘Lots of clients don't even know that practices have a complaints policy. They don't know that they have to follow that and they don't know how to find it […] and that's when they become frustrated and it can get messy’ (Mediator 1)
		People within practices	‘The practice was really good about it and the vet [name] was so kind, so lovely, explaining it all and encouraging me to talk about it with them. I was [swear] furious don't get me wrong, but feeling like they cared and being listened to helped’ (Client 4)
Motivation: cognitive, habitual and emotional processes that drive the behaviour	Emotional reactions	‒	‘I think a lot of clients when they phone us are frustrated with their practice but we do have a really wide variety of emotions that come through the service’ (Mediator 5) ‘It was so, so, so upsetting … it really was, yes the whole thing from start to finish if there ever was a finish. I'm still upset now’ (Client 3)
	Fairness perceptions	‘Now culture’	‘… sometimes they are aware of how to [pursue a complaint with the practice], they don't want to because they want it to be sorted NOW. They question the practice if they are taking even what is probably a normal amount of time to respond. This now culture is a big thing …’ (Mediator 1)
		Moral status of animals	‘He was my everything at that time … just because he was an animal it doesn't mean it didn't matter because if he was a person it would just not have been acceptable and they would have looked at it properly or differently I'm sure’ (Client 3)
		The ‘right’ to be compensated	‘they demand things … they say things like—I want compensating for what I've been through, it's only right and they believe that they have a right to it and often it's what it's about’ (Mediator 1)
		The duty of veterinarians	‘I mean, I have had clients say to me, I don't care if there's a recruitment crisis. You know, the practice shouldn't be open anyway, they haven't got enough staff. They should just close the practice. They don't get it. They just think about the duty the vets have’ (Mediator 1)
	Desiring an outcome	Prevention of future events	‘the client only wanted to know what the practice had done to make sure that that drug isn't prescribed again. And she didn't want any money or anything like that … she said “I'm not here to get anyone into trouble”… I just need to know for my own mind that they won't make that mistake again’ (Mediator 4)
		Financial redress	‘I have had some ridiculous amounts … £10,000 but also just a client saying give 100 pound donation to a charity’ (Mediator 1)
		Honesty	‘I wanted them to tell me exactly what happened and what went wrong and if they didn't know just to be part of an open dissection … or at least be able to look at the notes or something […] … it breeds distrust of people when it feels like there's cover ups’ (Client 1) ‘[mediator discussing the particulars of one clients’ expression of what they found helpful at the end of the process] … others, they are saying “the explanation was very helpful” even if the client doesn't agree, like they are poles apart, they are happier with knowing about it’ (Mediator 1)
		Apology	‘often it goes like … I just want them to say sorry, you know … I understand there's a mistake that's being made’ (Mediator 1)
		Accountability	‘… they do tend to blame the vets a lot I have to say’ (Mediator 3) ‘they'll be like … “I want the vet struck off” Well we have to explain it isn't going to happen here’ (Mediator 1)

#### Capability

Having previous experience of complaints either in the veterinary sector or elsewhere was discussed. Participants’ knowledge of complaint processes in the veterinary or other sectors and prior experience in a personal or professional context were mentioned. While participants recognised the benefits of technological factors for communicating dissatisfaction in relation to adverse events, lack of personal ability and/or familiarity in using such channels was mentioned as a barrier to relaying concerns. Interestingly, clients’ self‐confidence and belief in their personal ability to comprehensively share their feelings, perspectives and opinions with the practice was raised by mediators but not by clients.

#### Opportunity

Clients’ complaint behaviour following adverse events was influenced by broad social and societal factors. The instant availability of information and a perceived rise in internet search engine use were specifically discussed. Concern about widespread exposure to advertising regarding clinical negligence claims and misconceptions regarding applicability to the veterinary sector was voiced. Both online and in‐person social networks impacted the nature of complainants’ behaviour. Friends and/or family members proposed that a complaint should be raised in the aftermath of an adverse event in many cases. Word‐of‐mouth and the ‘grapevine effect’, accelerated by the use of social media, were specifically referenced. Support and encouragement provided by others was mentioned, as was the immediacy with which this often happened. Organisational factors, including veterinary practice processes, were identified as equally important facilitators shaping complaint behaviour. A potential barrier to constructive complainant engagement was clients’ perceptions of a lack of availability of informal organisational feedback channels. Frustration brought on by confusion regarding complaint processes was also described and suggested as a reason for clients escalating complaints beyond a practice level. People within practices were highlighted to have both positive and negative influences on the nature of client complaint behaviour. Practitioner approachability and compassion were thought to mitigate heightened emotional states among clients and represented an opportunity for practitioners to divert negative client complaint behaviour.

#### Motivation

Emotional responses were commonly discussed by both clients themselves and mediators, with a negative emotional state being described as underpinning many clients’ behavioural reactions to adverse events. Perceptions of fairness of the adverse event outcome and the procedural and interpersonal dimensions of the response in the aftermath were alluded to by both groups of interviewees. The perceived unfair duration between the adverse event and resolution of the emotional distress experienced was discussed in the context of a wider ‘now culture’. The lack of established moral status of animals was highlighted by the clients, who mentioned that they perceived that adverse events involving animals were not treated in the same way as similar events in a human healthcare context. Clients’ beliefs and expectations regarding redress and a right to monetary compensation and the duty that veterinary practitioners and organisations owe clients and broader society were raised by mediators and clients. Desiring an outcome was a large category, derived from five subcategories reflecting a range of clients’ desires, and was discussed by all participants. The clients interviewed seemed to be partly motivated by altruistic tendencies in their sentiment of preventing future events. Some recognised that they may be able to draw attention to the support needs of individual practitioners. Although mediators voiced a perception that clients often lacked regard for the emotional and professional wellbeing of the practitioners involved, they pointed out that clients were keen to suggest the role that practitioner education and learning may have in preventing future adverse events. Preventing future clients from experiencing similar events was a clear focus for some client interviewees, a notion backed up by the experiences of mediators. Acknowledgement of patient safety concerns by others was implied to be important, and a desire for both verbal and written assurance that measures would be taken to prevent the same or similar happening in the future was voiced. Although the desire for financial redress was discussed in all the interviews, expectations regarding acceptable levels of monetary reward were wide ranging. Some participants were eager to highlight that it was not greed but principle that drove a desire for financial redress. Suggestions for charity donation, rather than personal gain, were mentioned by a few mediators.

Calls for honesty and transparency in the wake of adverse events were brought forward by clients who alluded that not knowing the truth has implications for the veterinarian‒client relationship. The mediators perceived that the mediation process was helpful in fostering such honesty and transparency and supported the notion that clients feel more satisfied when they feel that they understand events, even if they disagree with the outcome or reasoning behind it. All the clients interviewed expressed a want to be supported and treated compassionately by both individual veterinary practitioners and organisations regarding their experiences of adverse events. They suggested that a central component of this rested in a simple heartfelt apology, not only acknowledging any veterinary patient harm but also the emotional distress they had experienced as a result. Interestingly, a desire for accountability of practitioners involved in adverse events was mentioned by all of the mediator participants. They described clients’ desire for the involved practitioners to be professionally sanctioned in relation to events. This was against a backdrop of the mediators discussing their own role in setting expectations regarding the mediation process.

#### Part 2: Quantification of clients’ desired outcomes in the aftermath of adverse events

**FIGURE 2:**
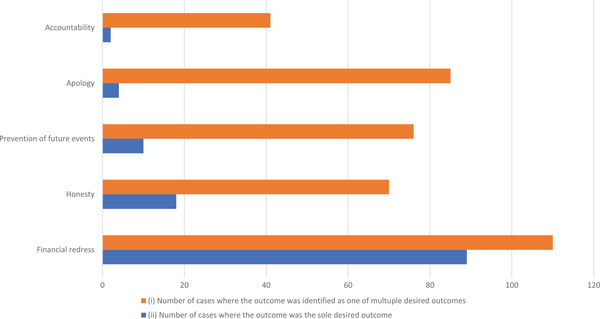
Number of resolved Veterinary Client Mediation Service cases (out of a total of 280) where each desired outcome was identified as (i) one of multiple desired outcomes and (ii) the sole desired outcome

**FIGURE 3:**
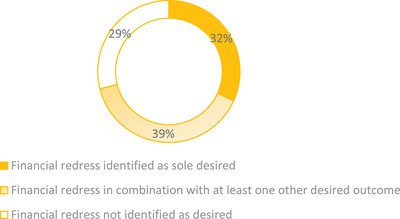
Proportion of 280 resolved Veterinary Client Mediation Service cases where financial redress was identified as a desired outcome by clients

A total of 381 resolved VCMS case records were reviewed. One hundred and one (26.5%) did not articulate the occurrence of an adverse event and/or lacked information regarding the desired outcome of the client and were excluded, leaving 280 records (73.5%) remaining. These 280 were coded using the subcategories of desired outcomes generated in part 1. A single category of desired outcome was most commonly identified (123/280; 43.9%), followed by a combination of two categories of desired outcome (96/280; 33.9%). In 199 of the 280 cases (71.1%), financial redress was identified as one of the desired outcomes, with honesty, prevention of future events and apology identified in a similar number of cases: 88 (31.4%), 86 (30.7%) and 89 (31.8%), respectively. Accountability was identified as one of the desired outcomes in 43 of the 280 cases (15.3%), making it the least frequently identified outcome. Although financial redress was the most commonly identified desired outcome (Figure 2), it was not identified (81/280 = 28.9%) or was identified only as one of multiple desired outcomes (110/280 = 39.2% of cases) in 191 of the 280 cases (68.2%) (Figure 3).

## DISCUSSION

To the authors' knowledge, this is the first study to provide insight into factors that may influence the nature of veterinary client complaint behaviour in the aftermath of adverse events, specifically from the perspective of clients and veterinary client mediators. The findings suggest that the nature of client complaint behaviour is influenced by a complex interplay of client capabilities, opportunities and motivations. Although clients commonly desire financial redress in the aftermath of adverse events, honesty, apology and efforts to prevent future occurrences were also shown to be valued. These insights may be used to inform the development of interventions that channel constructive rather than detrimental client complaint behaviours.

A number of capabilities and opportunities were identified as influencing the nature of veterinary client complaint behaviour in this study. The findings reflect an abundance of literature from the human healthcare sector, which suggests that complaint behaviour among patients and their families is influenced by their ability to access online information^49^, ^50^ and their knowledge of complaint processes.^51^ Capabilities, and some opportunities, are inherent to individuals. They include, but are not limited to, those created by structural and social norms. Organisations may have direct and indirect influence over complainants’ capabilities and opportunities to constructively complain. This may be achieved by increasing accessibility to complaint processes. In doing so, organisations may reduce unfounded complaint escalation and seize opportunities to learn and reconcile differences in the wake of dissatisfaction. In recognition of this, attempts to transform hospital complaints handling have seen accessibility prioritised.^52^ In human healthcare, one mechanism utilised is collaboration with community outreach and advocacy groups.^53^ Further research would be needed to gauge both the appetite for signposting to such external services by veterinary care providers and the benefits of doing so. More intuitive suggestions for influencing clients’ capabilities and for providing opportunities to constructively complain can be translated from human healthcare. These include improving the comprehensibility of information surrounding complaint processes and ensuring that a diverse range of complaint channels are available.^54^ Reducing complainant stigmatisation is also likely to be important. Human healthcare patients report an increased readiness to provide feedback where they do not feel ostracised and/or are encouraged to do so by practitioners providing their care.^55^

Clients’ desire for practices to be honest about factors that may have contributed to adverse events, to receive an apology and for measures to be taken to prevent similar future occurrences were also identified as key factors motivating the nature of their complaint behaviour in this study. The findings resonate with recent work by Gordon et al., where concealment of mishaps was concluded to underpin some clients’ drive to complain to the Veterinary Council of New Zealand.^56^ Comprehensive veterinary case records, which veterinary practitioners are not only encouraged to write but are a component of their professional code of conduct,^57^ may provide a useful defence against complainants’ accusations in the aftermath of adverse events.^58^ However, they do not allow for nuanced explanations of the circumstances that led to an event, an apology or reassurances that organisations are attempting to prevent similar occurrences in the future. These factors were all highlighted by the clients in this study. Adverse event review processes bestow an opportunity to explore factors that contribute to events and to make changes that prevent future occurrences. Despite becoming embedded in veterinary practices,^59^, ^60^ inclusion of veterinary clients’ voices in such processes is neither suggested by the regulator nor formally embraced at a practice level. Given the findings here, such a move would be worthy of future consideration.

In contrast, patient and family engagement is a key priority following adverse events in human healthcare. It was identified as a theme within a recent study that explored the desires of patients and their families in relation to human healthcare adverse event review processes.^61^ The findings of the study also highlighted the value of demonstrating improvements and acknowledging emotional harm to patients in the wake of adverse events. Patient‐centred approaches to adverse event review reflect a broader paradigm shift away from paternalism, where practitioners assume a superior knowledge of what is best for patients and hence direct diagnostics and treatment decisions, towards patient‐centred healthcare delivery. Patient centredness^62^, ^63^ recognises that understanding and respecting the perspectives of patients and their families underpins practitioners’ and organisations’ ability to prevent and reconcile conflict, to mitigate emotional harm and to ultimately optimise the accessibility, safety and quality of care provided.^64^, ^65^ By fostering collaborative relationships between those providing and receiving care and promoting seamless multidisciplinary working, patient‐centred approaches are additionally thought to enhance practitioners’ sense of workplace satisfaction.^66^

Client centeredness, the equivalent concept in veterinary healthcare, is gaining traction,^67^, ^68^ particularly in light of shifting perspectives and knowledge surrounding animal ownership, the role that the human‒animal bond plays in mental health^69^, ^70^, ^71^ and acknowledgement that contextualised care is necessary.^72^, ^73^, ^74^ Client centredness is of particular interest against the backdrop of veterinary organisational consolidation, technological advances, surges in animal ownership^75^, ^76^ and concerns regarding the accessibility and affordability of care. Although solicitation of clients’ perspectives during the care process has been proposed as an important factor in reducing the likelihood of veterinary malpractice claims,^77^, ^78^ to the authors’ knowledge, this is the first time client engagement in the aftermath of adverse events has specifically been explored from the perspective of clients.

This study indicates that fairness perceptions and emotional reactions influence the nature of veterinary client complaint behaviour. This aligns with consumer behaviour theory, which suggests that customers are more likely to react negatively when they perceive injustice^13^ and that organisations may reduce perceived injustice by proactively initiating consumer voice behaviour.^79^ The findings in this study additionally prompt questions regarding the extent to which it is appropriate to engage with clients during review of adverse events and the barriers and facilitators to doing so. A Canadian‐based study evaluating communication during animal euthanasia discussions concluded that a discrepancy may exist between veterinary practitioner and client evaluations of client centeredness.^80^ Further to the current study, a similar comparative exploration of veterinary client and practitioner perceptions of client centredness in the aftermath of adverse events would be of interest. Combined with the findings here, such a study could act as a starting point for further research that addresses whether training and support interventions aimed at refining and refocusing veterinarian‒client communication efforts following adverse events are necessary.

This study provides qualitative insight into clients’ motivating desire for financial redress in the aftermath of adverse events and quantitatively suggests that financial redress is the most common desired outcome of veterinary client complainants. In light of the scrutiny the profession is currently facing regarding consumer experience,^81^, ^82^ this study raises timely questions about what is economically appropriate when there is real or perceived veterinary service failure. Despite financial redress being identified as a key influencer of client complaint behaviour in the VCMS cases, in more than two‐thirds of cases, financial redress was either not identified or was identified as only one of a number of desired outcomes. The findings thus suggest that the nature of veterinary clients’ complaint behaviour is influenced by a complex of motivating desired outcomes rather than solely financial reasons. It is not possible to draw conclusions from the findings here about whether achieving one desired outcome influences clients’ desire to achieve another. Associations between certain types of negative complaint behaviour and desired outcomes cannot be made either. However, the findings, suggest that veterinary organisational responses to adverse events must consider a multitude of client desires if damaging escalation of complaint behaviour is to be prevented.

### Limitations

The interview technique used in this study allowed veterinary clients to express feelings and opinions about complaint behaviours in relation to adverse events in their own words, allowing a rich understanding to be developed. However, the sample was small and the convenience technique used to recruit participants introduces selection bias. Interviews with veterinary client mediators, along with analysis of mediation records, allowed a broader and more impartial understanding to be developed. Mediation records analysed were not collected solely for the purpose of this research and represent a naturalistic data source. Although such data sources are not affected by social desirability bias (as interviews may be), retrospective review is impacted by researcher interpretation as well as being restricted in scope when data are incomplete. This study used a subset of mediation records, previously categorised as ‘standard of care’ issues by mediators, and the findings are therefore affected by mediators’ interpretations. Although quantification of qualitatively derived desired outcomes stimulates hypotheses regarding those most likely to influence client complaint behaviour, figures here are descriptive only. Larger sample sizes collected using a random sampling technique would be needed to test statistical significance. The interview data collected from mediators and documented mediation records provide a specific window through which to understand client complaint behaviour. The perspective of clients who do not pursue mediation, have their concerns resolved prior to involvement of mediation, are not amenable to mediation and/or prefer adversarial channels are not included. Although these factors mean that the findings cannot be generalised to populations beyond this specific data set, they represent an introductory examination of the relevant issues. It would be interesting to explore whether mediation is pursued more commonly by different types of veterinary client and/or following certain types of adverse event.

### Recommendations and Future Research

The following methods should be explored to reduce the likelihood of negative veterinary client complaint behaviour and to harness client complaint information for quality improvement purposes.

Veterinary practices should reduce stigma surrounding client complaints by encouraging practitioners to share their experiences with colleagues and to engage with alternate dispute resolution processes where appropriate. Development of guidelines for practitioners to use when interacting with clients following adverse events may be warranted. Regular review of complaint processes to ensure that information provided to clients is up‐to‐date, comprehensive and accessible would also be beneficial.

Research to develop complaint coding taxonomies is worthy of investment. These could be used as a base for centralised informatics systems that are used to store, analyse and triangulate complaint data against adverse event reporting data and quality metrics. The development of such systems may be facilitated by the RCVS and/or professional indemnity and animal health insurers.

### Conclusion

The nature of client complaint behaviour is influenced by a complex of factors, some of which may be amenable to organisational intervention. Although suggesting that clients have a propensity to seek financial redress in the aftermath of adverse events, the findings of this study highlight the value of organisational efforts to learn and improve as a result; for at least a proportion of complainants, the desire for honesty, evidence of efforts to prevent future events and apologies, wholly or in part, was demonstrated to drive pursuance of complaints. Proactively engaging with clients in relation to adverse events should be considered in veterinary quality improvement initiatives, but more research is needed to establish how best this can be achieved.

## AUTHOR CONTRIBUTIONS

Julie Gibson conducted the study and wrote the manuscript. Kate White, Marnie L. Brennan and Liz Mossop contributed to the design of the study, discussed the analysis and results and reviewed iterations of the manuscript.

## CONFLICT OF INTEREST STATEMENT

The authors declare they have no conflicts of interest.

## ETHICS STATEMENT

Ethical approval for the study was granted by the School of Veterinary Medicine and Science's Committee for Animal Research and Ethics at the University of Nottingham (approval numbers 3892 230725, 2444 180724 and 3790 230215).

## Data Availability

Transcripts and mediation records contain information that may compromise the anonymity of contributors and are therefore not available to be shared.
